# Serum PCB levels and congener profiles among teachers in PCB-containing schools: a pilot study

**DOI:** 10.1186/1476-069X-10-56

**Published:** 2011-06-13

**Authors:** Robert F Herrick, John D Meeker, Larisa Altshul

**Affiliations:** 1 Department of Environmental Health, Harvard School of Public Health, 665 Huntington Ave., Boston, MA 02115, USA; 2 Department of Environmental Health Sciences, University of Michigan School of Public Health, 109 S. Observatory St, Ann Arbor, MI 48109, USA

## Abstract

**Background:**

PCB contamination in the built environment may result from the release of PCBs from building materials. The significance of this contamination as a pathway of human exposure is not well-characterized, however. This research compared the serum PCB concentrations, and congener profiles between 18 teachers in PCB-containing schools and referent populations.

**Methods:**

Blood samples from 18 teachers in PCB-containing schools were analyzed for 57 PCB congeners. Serum PCB concentrations and congener patterns were compared between the teachers, to the 2003-4 NHANES (National Health and Nutrition Examination Survey) data, and to data from 358 Greater Boston area men.

**Results:**

Teachers at one school had higher levels of lighter (PCB 6-74) congeners compared to teachers from other schools. PCB congener 47 contributed substantially to these elevated levels. Older teachers (ages 50-64) from all schools had higher total (sum of 33 congeners) serum PCB concentrations than age-comparable NHANES reference values. Comparing the teachers to the referent population of men from the Greater Boston area (all under age 51), no difference in total serum PCB levels was observed between the referents and teachers up to 50 years age. However, the teachers had significantly elevated serum concentrations of lighter congeners (PCB 6-74). This difference was confirmed by comparing the congener-specific ratios between groups, and principal component analysis showed that the relative contribution of lighter congeners differed between the teachers and the referents.

**Conclusions:**

These findings suggest that the teachers in the PCB-containing buildings had higher serum levels of lighter PCB congeners (PCB 6-74) than the referent populations. Examination of the patterns, as well as concentrations of individual PCB congeners in serum is essential to investigating the contributions from potential environmental sources of PCB exposure.

## Background

Although PCBs have been banned from commerce since the late 1970s in most of the world, they persist in many environmental settings. Recent interest in building materials as possible sources of PCB contamination in the built environment has led to a series of investigations, and a set of EPA guidance documents on "PCBs in Caulk in Older Buildings" [1]. While evidence for contamination of indoor air from building materials has been growing recently, its significance as a possible source of PCB exposure to humans living or working in these buildings is less clear. Several investigations have provided evidence that occupancy of buildings containing PCB-rich building materials can result in elevated serum PCB levels. The investigation of indoor contamination resulting from PCB-containing caulking (sealant) material first reported by Benthe et al. [2] found elevated indoor air levels of the less-chlorinated PCB congeners 28, 52, and 101, however elevated serum levels were not observed among the building occupants. More detailed investigations confirmed the importance of building caulking and sealing materials as diffuse sources of PCB contamination [3,4]. Gabio et al. [5], and Schwenk et al. [6] reported elevated PCB indoor air concentrations (congeners 28, 52 and 101) in schools containing PCB caulking materials. Teachers in these buildings had serum levels of PCB 28, 52, 101, 153 and 138 up to eight times greater than age-matched comparison subjects. Johansson et al. [7] reported a study of PCB-containing residential apartment buildings in which indoor air concentrations of PCB (sum of congeners 28, 52, 101, 138, 153, and 180) were found to be up to twice the levels measured in similar buildings without such sealants. Residents of the PCB-containing buildings showed significant elevations for serum levels of PCB 28, 74, 66 and 99, and also for total PCB based on the sum of 30 congeners. Liebel et al. [8], compared 377 children attending a PCB-containing school with 218 attending an uncontaminated school and reported that at least one of the lower chlorinated PCB congeners (of the six WHO-indicator congeners PCB 28, 52, 101, 138, 153, 180) could be detected in the blood of all the students at the PCB-containing school, compared to 27% of the students at the comparison school. Significantly higher median serum concentrations for PCBs 28, 52, and 101were found in the students from the contaminated school, compared to the students from the control school. There was a significant positive association between years spent at the contaminated school and serum levels of the combined lower chlorinated congeners.

The overall goal of the current study was to evaluate the possible relationship between serum PCB concentrations, and employment in schools containing PCB in building materials. Specifically, our research aims included comparisons of total serum PCB concentrations between teachers who worked in these buildings and available reference groups who did not work in these buildings, as well as examination of the composition of the congener mix found in the serum of the teachers and the referents.

## Methods

A convenience sample of 18 teachers who worked in 3 schools containing PCB caulking material was recruited from current members of a local labor union. These three schools were constructed during the 1960s, corresponding to a period when PCB caulking was used in masonry buildings. The presence of PCB in the caulking of these schools was confirmed by testing conducted by the teachers' union. The subjects were volunteers recruited by the investigators from a population of approximately 3,000 members of the teachers' union. In addition to collecting a blood sample, the teachers were interviewed by the investigators (RH) about their work history, diet, smoking, and history of pregnancies and breast feeding. A nonfasting venous blood sample was collected from the 18 volunteers on the last day of the school year in June 2009. Blood samples were centrifuged and serum stored in glass Wheaton vials at -20°C until analysis. Measurement of PCBs, p,p'-DDE and HCB in serum was conducted by the Organic Chemistry Analytical Laboratory, Harvard School of Public Health, Boston, MA. Target analytes included 57 individual PCB congeners, p,p'-DDE, and HCB. This set of target congeners included more low chlorinated (more volatile) congeners than are measured in the set of 35 NHANES congeners, because the methods used by our laboratory are applied to analyze air samples, as well as biological samples. Serum extracts were analyzed by dual capillary column gas chromatography with electron capture detection (GC/ micro ECD) and quantified based on the response factors of individual PCB congeners relative to an internal standard [9]. All final concentrations were reported after subtracting the amount in procedural blanks associated with the analytic batch. Results were not adjusted by surrogate recoveries. Wet weight serum PCB concentrations (e.g. ng/g serum), as well as lipid adjusted serum levels, were reported. Details of the quality assurance and quality control procedures are provided in Additional File 1: Quality Assurance and Quality Control.

This study was approved by the Office of Human Research Administration at the Harvard School of Public Health.

### Data analysis

We used several approaches to examine the results of the serum analysis. First, we compared serum levels and congener patterns within the group of 18 teachers. We also compared the results from the teachers with the National Health and Nutrition Examination Survey (NHANES) participants. Our laboratory and the NHANES data report 33 of the PCB congeners in common. The primary difference between these laboratories is that of the 57 congeners our lab reports, 20 congeners are in the range of di-, tri- and tetrachloro congeners (IUPAC # 6-74), while the NHANES data reports only 6 in this range of lighter congeners.

The third comparison was between the teachers, and the results of serum PCB determinations from 358 men who were seeking infertility diagnosis from the Vincent Burnham Andrology lab at Massachusetts General Hospital (MGH) in Boston (January 2000 - May 2003). These are referred to as MGH referent samples [9]. These men were chosen as referents because their samples were run in the same lab as the teachers', using the same methods, their age ranges overlapped with the teachers, and they resided in the Greater Boston area (as did the teachers). The Harvard laboratory that analyzed the samples from the 18 teachers, and the MGH referents reported results for the same 57 PCB congeners.

We compared the serum levels of whole-weight and lipid adjusted congeners between the teachers and the referent groups, and examined the patterns of congeners between the groups. Finally, to further assess potential differences in congener patterns between groups, the composition of the congener mix in these 18 teachers was compared with the MGH referent subjects by principal components analysis (PCA). All measured congeners were normalized to PCB 153 and transformed by the natural logarithm prior to PCA analysis to obtain a unitless profile matrix as described previously [10].

## Results

### Characteristics of the teacher study subjects

The teachers ranged in age from 33 to 64 years. All had worked in their current school for at least 6 years, all were non-Hispanic whites, and the majority (13 of 18) were women. The subjects are described in Additional File 2: Characteristics of teacher study subjects. Examining the trend in serum levels with age, we found that there was a strong correlation for the sum of whole-weight values of the 57 PCB congeners (Pearson correlations, ln-transformed PCB concentrations R = 0.84), and for the heavy PCBs (congeners 84-209, R = 0.84). The levels of lighter (congeners 6-74) PCBs were much less strongly correlated with age, R = 0.49. This age related trend was also observed for lipid-adjusted values (sum of 57 PCBs R = 0.75; heavy congeners R = 0.77, light congeners R = 0.35).

Within the group of 18 teachers, we observed distinct differences in the patterns of serum congener profiles. Seven of the 10 teachers from School A had serum levels of lighter (PCB 6-74) congeners that exceeded the median for the entire group of 18 (0.24 ng/g whole weight). These seven included several of the younger (< age 50) teachers who had some of the lowest total (sum of 57 congeners) serum levels. These lighter congeners, therefore, made a much greater contribution to the overall serum PCB levels for these subjects than for the other teachers (Table 1). For three of the younger teachers at School A, this elevated fraction of light congeners appeared to be driven by disproportionately high levels of PCB 47.

**Table 1:** Teachers' whole weight serum PCB concentrations, % light congeners, and congener ratio

Subject	Age	School	TotalPCB^1^	Light PCB^2^	Light PCB %total	PCB47^1^	PCB47% total	Ratio PCB 47:153
12	35	A	0.74	0.24	32.29	0.10	12.97	1.22
8	41	A	0.58	0.21	35.63	0.14	23.28	1.96
11	41	A	1.09	0.30	27.40	0.16	14.33	1.00
16	46	A	1.49	0.17	11.38	0.01	0.70	0.05
15	47	A	1.42	0.28	19.96	0.08	5.90	0.34
4	48	A	2.02	0.28	14.09	0.10	5.18	0.33
3	49	A	0.96	0.18	18.72	0.04	4.32	0.25
10	54	A	1.92	0.30	15.54	0.12	6.45	0.33
17	56	A	2.45	0.29	11.79	0.08	3.20	0.18
1	62	A	1.88	0.29	15.38	0.07	3.90	0.22
6	33	B	0.80	0.17	20.89	0.01	1.36	0.08
9	37	B	0.73	0.13	17.54	0.01	1.87	0.12
2	56	B	1.38	0.12	8.95	0.02	1.56	0.09
14	59	B	1.68	0.24	14.55	0.08	4.64	0.28
5	52	C	2.46	0.20	8.11	0.03	1.02	0.06
7	60	C	2.17	0.20	9.17	0.02	1.04	0.05
13	62	C	4.37	0.40	9.20	0.02	0.46	0.02
18	64	C	5.32	0.71	13.29	0.03	0.05	0.03

^1^ng/g serum

^2 ^ng/g serum, light PCB congeners IUPAC 6-74

### Comparison with 2003-4 NHANES data

The Harvard Organics Laboratory that analyzed the 18 teachers' samples, and the 2003-4 NHANES reported serum levels for 33 of the same PCB congeners. The comparison of the 18 teachers with the age-stratified NHANES data only for the 33 congeners they report in common is presented in Table 2. Non-parametric statistical analysis comparing the sum of 33 congeners for the 18 teachers with the same age group (33-64 years) of NHANES subjects (Wilcoxon rank sum test) showed no difference between the groups overall (teacher median = 1.32 ng/g, NHANES median = 1.11 ng/g, p = 0.25), while the older teachers (age 50-64) had significantly higher levels than the age-comparable NHANES subjects (teacher median = 2.14 ng/g, NHANES median = 1.49 ng/g, p = 0.05). Teachers who reported more than one meal of dark fish or liver per week (subjects 5 and 18) had serum levels well above the NHANES GM for their age groups.

**Table 2:** Comparison of teachers with age-stratified NHANES levels for 33 PCB congeners common to both datasets, non-Hispanic whites

Subject by age	School	∑33 NHANES congeners ng/g serum (pg/g lipid)	NHANES GM	NHANES 90%	NHANES 95%
**20-39**			**0.47(79.2)**	**1.03(157)**	**1.47(226)**
**6**	B	0.69(118)			
**12**	A	0.55(158)			
**9**	B	0.64(172)			
**40-59**			**1.21(186)**	**2.48(375)**	**3.22(471)**
**8**	A	0.38(106)			
**11**	A	0.83(147)			
**16**	A	1.38(240)			
**15**	A	1.23(268)			
**4**	A	1.79(214)			
**3**	A	0.82(161)			
**5**	C	2.30(391)			
**10**	A	1.67(270)			
**17**	A	2.22(342)			
**2**	B	1.25(228)			
**14**	B	1.51(183)			
**60+**			**2.27(347)**	**4.31(689)**	**5.91(929)**
**7**	C	2.03(364)			
**1**	A	1.66(268)			
**13**	C	4.16(694)			
**18**	C	4.98(1346)			

Examining the 6 congeners both laboratories reported in the range of lighter congeners (PCBs 28, 44, 49, 52, 66, 74), there was no significant difference (teacher median = 0.10 ng/g, NHANES median = 0.12 ng/g, p = 0.09) in serum concentrations. Comparing the percent contribution of the light congeners to the total (sum of 33 congeners) serum PCB level, the teachers' values were significantly lower than the NHANES values (teachers 8.2%, NHANES 11.3%, p = 0.005). These same patterns of differences were apparent when comparing lipid adjusted serum levels (Table 2).

### Comparisons with MGH referent subjects

Comparing total serum PCB concentrations (sum of 57 congeners) between the 18 teachers with the 358 MGH referent subjects, 12 of the 18 exceeded the median level of total PCBs for the MGH referents, and 4 exceeded the upper 95% level for these referents. The oldest MGH referents were 51 years old, so we separately compared the 9 teachers younger than age 50 with the MGH referents. These younger teachers (under age 50) had total serum concentrations that were not different from the referents (teacher median = 1.01 ng/g, MGH median = 1.09 ng/g, p = 0.61, Wilcoxon rank-sum test). Comparing all 18 teachers with the MGH referents (Table 3), the teachers had somewhat higher total serum PCB levels overall (teacher median = 1.55 ng/g, MGH median = 1.09 ng/g, p = 0.02).

**Table 3:** Teacher to NHANES and MGH referent median ratios

PCB Cong-ener	18 teacher median (ng/g serum)	MGH referent median (ng/g serum)	Ratio 18 teachers:MGH referent	Teachers < 50 yrs median (ng/g serum)	Ratio teachers < 50 yrs:MGH referents	NHANES median subjects < 50 yrs old/ > 50 yrs (ng/g serum)	RHA^f^
**6^a^**	0.0017	0.0001	16.9931	0.0028	27.6006		0.001
**8^a,e^**	0.0071	0.0001	71.1125	0.0067	67.2269		0.001
**16 ^e^**	0.0038	0.00113	3.3367	0.0033	2.9499		0.005
**18 ^e^**	0.0033	0.00198	1.6906	0.0025	1.2732		0.003
**25^a^**	0.0012	0.0001	12.1379	0.0005	5		0.024
**26^a^**	0.0008	0.0001	7.7850	0.0008	8		0.016
**28^b,c,e^**	**0.0222**	**0.00757**	**2.9357**	**0.0223**	**2.9417**	**0.031/0.030**	**0.12**
**31 ^e^**	0.0023	0.00048	4.7290	0.0017	3.5014		0.019
**33^a,b,e^**	0.0185	0.0001	185.483	0.01600	159.663		0.011
**37^a^**	0.0040	0.0001	40.4865	0.0025	25.2101		0.07
**41^a^**	0.0016	0.0001	16.129	0.0009	8.7719		0.008
**44^d,e^**	**0.0034**	**0.00158**	**2.1274**	**0.0032**	**2.0416**	**0.013/0.012**	**0.009**
**47^a^**	0.0574	0.0068	8.4379	0.0836	12.2961		0.03
**49**	**0.0031**	**0.00057**	**5.5081**	**0.0023**	**4.0800**	**0.008/0.007**	**0.01**
**52^c,e^**	**0.0054**	**0.00254**	**2.1201**	**0.0048**	**1.8748**	**0.002/0.017**	**0.009**
**60^b, d^**	0.0029	0.0024	1.1942	0.0026	1.0776		0.06
**66^b,d,e^**	**0.0159**	**0.0042**	**3.7751**	**0.0106**	**2.5284**	**0.008/0.009**	**0.07**
**70 ^a,d,e^**	0.0070	0.00093	7.5341	0.0053	5.7094		0.05
**74^b^**	**0.0561**	**0.0331**	**1.6937**	**0.0364**	**1.1007**	**0.028/0.055**	**0.27**
**84**	0.0009	0.00034	2.5577	0.0009	2.5355		0.04
**87^a^**	**0.0032**	**0.0001**	**31.7540**	**0.0031**	**30.9734**	**0.005/0.005**	**0.016**
**95 ^e^**	0.0089	0.0042	2.1207	0.0103	2.4565		0.01
**97^a^**	0.0052	0.0001	52.1779	0.0047	46.5116		0.06
**99**	**0.0444**	**0.0344**	**1.2923**	**0.0377**	**1.0959**	**0.024/0.036**	**0.28**
**101^e^**	**0.0173**	**0.0046**	**3.7594**	**0.0133**	**2.89271**	**0.010/0.009**	**0.03**
**105**	**0.0142**	**0.007**	**2.0222**	**0.0082**	**1.1699**	**0.006/0.010**	**0.32**
**110^d^**	**0.0081**	**0.0035**	**2.3041**	**0.0078**	**2.2167**	**0.007/0.007**	**0.014**
**118**	**0.0795**	**0.0595**	**1.3369**	**0.0613**	**1.0301**	**0.031/0.059**	**0.5**
**128**	**0.0029**	**0.0008**	**3.6754**	**0.0025**	**3.1512**	**0.001/0.001**	**0.3**
**135**	0.0042	0.0015	2.7809	0.0043	2.8736		0.02
**136^a^**	0.0043	0.0001	43.1323	0.00420	42.0168		0.008
**138**	**0.1527**	**0.158**	**0.9666**	**0.0798**	**0.5053**	**0.097/0.178**	**0.6**
**141**	0.0024	0.007	0.3456	0.0018	0.2506		0.06
**146**	**0.0353**	**0.0215**	**1.6404**	**0.0189**	**0.8794**	**0.013/0.027**	**1.6**
**149^a^**	**0.0133**	**0.00016**	**83.329**	**0.0142**	**88.4956**	**0.004/0.004**	**0.04**
**151**	0.0073	0.0015	4.8602	0.0070	4.6784	0.002/0.002	0.03
**153**	**0.2611**	**0.2016**	**1.2953**	**0.1561**	**0.7745**	**0.132/0.251**	**1.1**
**156**	**0.0407**	**0.0272**	**1.4973**	**0.0140**	**0.5156**	**0.022/0.044**	**2.3**
**157**	**0.0109**	**0.0084**	**1.2961**	**0.0061**	**0.7310**	**0.005/0.010**	**1.1**
**167**	**0.0121**	**0.0074**	**1.6395**	**0.0079**	**1.0669**	**0.004/0.009**	**1.2**
**170**	**0.0806**	**0.06**	**1.3430**	**0.0368**	**0.6140**	**0.042/0/081**	**2**
**171**	0.0106	0.0061	1.7353	0.0059	0.9643		0.7
**174^a^**	0.0078	0.0001	77.771	0.0071	70.7965		0.024
**180**	**0.2278**	**0.142**	**1.6045**	**0.1067**	**0.7516**	**0.117/0.229**	**2.9**
**183**	**0.0280**	**0.0172**	**1.6255**	**0.0151**	**0.8788**	**0.010/0.019**	**0.5**
**187**	**0.0595**	**0.0445**	**1.3362**	**0.0293**	**0.6587**	**0.028/0.057**	**1.4**
**189**	0.0036	0.0029	1.2403	0.0009	0.3025	0.001/0.001	2.6
**194**	**0.0633**	**0.03**	**2.1090**	**0.0198**	**0.6583**	**0.026/0.056**	**3.1**
**195**	**0.0104**	**0.007**	**1.4849**	**0.0042**	**0.6002**	**0.006/0.012**	**1.1**
**196**	**0.0329**	**0.016**	**2.0590**	**0.0176**	**1.1029**	**0.022/0.042**	**0.8**
**199**	**0.0442**	**0.031**	**1.4271**	**0.0167**	**0.5376**	**0.023/0.053**	**0.04**
**203**	0.0399	0.016	2.4930	0.0146	0.9159		1.8
**206**	**0.0257**	**0.016**	**1.6070**	**0.0113**	**0.7090**	**0.014/0.032**	**2.1**
**209**	**0.0086**	**0.0067**	**1.2903**	**0.0043**	**0.6433**	**0.007/0.020**	**1.8**
**201/177**	**0.01210**	**0.0093**	**1.3007**	**0.0101**	**1.0843**	**0.008/0.016**	**2.9/1**

Legend: Superscripts refer to congeners that have been identified as markers of occupational or environmental, non-dietary PCB exposures. Congeners included in the NHANES data are in **bold font**.

Congeners identified as markers of non-dietary exposure

^a^Herrick, et al., 2007

^b^Loutano, et al., 1991

^c^Kontas, et al., 2004

^d^Wingfors, et al., 2008

^e^DeCaprio, et al., 2005

^f^Relative Human Accumulation factors, Brown, 1992 (Ref 13)

**Figure 1:**
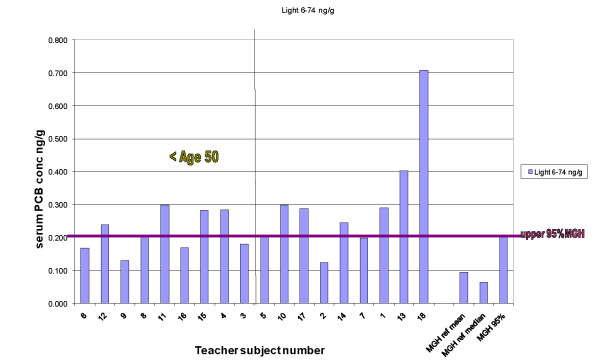
**Serum concentrations of light congeners (PCB 6-74), teachers compared to MGH referent values**.

We also compared the concentrations of lighter congeners (di-, tri- and tetrachloro, PCB 6-74) in the 18 teachers with the 358 MGH referents. These include less-persistent congeners that are more likely to volatilize from building materials than the heavier congeners (PCB 84-209). For the sum of congeners 6-74, all 18 teachers exceeded the MGH referent median, and 11 exceeded the upper 95% level for the MGH referents, including 5 of the 9 teachers aged 50 and younger (Figure 1). Statistical analysis by the Wilcoxon rank-sum test showed the teachers of comparable age to the MGH referents (50 and younger) to have significantly higher (teachers < 50 years median = 0.20 ng/g, MGH median = 0.08 ng/g, p < 0.0001) light PCB concentrations compared to the MGH referents. This significant difference was seen for the entire group of 18 as well (all teachers median = 0.23 ng/g, MGH median = 0.08 ng/g, p < 0.0001). These same differences were found when comparing the lipid-adjusted values as well.

The contribution of the lighter congeners (PCB 6-74) to the total serum level for each of the 18 teachers was compared to the MGH referent group. All 18 teachers exceeded the median % contribution of lighter congeners to the total for the MGH referents, by as much as a factor of approximately 5 (Table 1). The percent contribution of light congeners to the total serum PCB level was significantly higher for those aged 50 and younger (teachers < 50 years median = 18.7%, MGH median = 7.2%, p < 0.0001) as well as for the entire group of 18 teachers (all teachers median = 14.5%, MGH median = 7.2%, p < 0.0001).

### Comparison of PCB serum congener profiles

The composition of the mix of PCB congeners between the teachers and the referents was also examined. The first comparison of PCB congener profiles between teachers and MGH referents was to examine the ratios of the congener-specific group median concentrations. Because of the age differences between the 18 teachers and the MGH referents, we separated the 9 teachers who were under 50 from those over 50 and compared them with the MGH referents (Table 3). Median concentration ratios of 10 or greater between teachers less than age 50 and the MGH referents were found for two dichloro-PCBs (6 and 8), two trichloro-PCBs (33 and 37), one tetra-PCB (47), two penta-PCBs (87 and 97), two hexa-PCBs (136 and 149), and one hepta-PCB (174). The teacher to referent median ratios did not differ substantially between the group of 18 teachers, and the subgroup of 9 below the age of 50.

**Figure 2:**
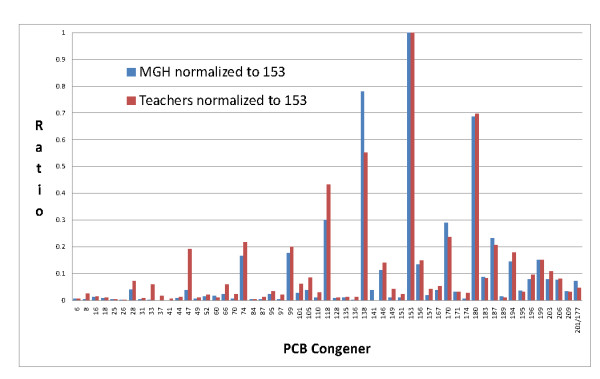
**Comparison of median serum congener concentrations normalized to congener 153 concentration between teachers and MGH referents**. Comparison of the height of the two bars (teachers and referents) for each congener illustrates the differences in the relative abundance of each congener in the serum of the teachers and the referents. The greatest differences are apparent in the lighter congeners, PCB 6-74, where the teachers' values consistently exceed the referents.

For 15 of the 18 teachers and all the MGH referents, the most prevalent congener found in serum was PCB 153. Calculating the ratios of the group median serum concentration for each congener to the group median concentration of congener 153 has the effect of normalizing the concentration of each PCB congener to the typically most prevalent congener [10]. For example, the ratio of the 18 teachers' median concentration of PCB congener 6 to the median concentration of congener 153 for the teachers can be compared to the same ratio in the MGH referents. A strength of this comparison is that it helps visualize the PCB composition for the subjects in each group, but it is insensitive to the absolute concentration of PCB in each sample. Comparing the congener-specific concentration ratios to PCB 153 between the 18 teachers and the MGH referents, substantial differences can be seen, a factor of 5 or more for PCBs 8, 33, 37, 41, 47 and 136. Another 13 congener ratios were at least twice as high in the 18 teachers compared to the referents. Most of these were in the range of lighter congeners (PCB 6-74, Figure 2). Three of the subjects had serum levels of congener 47 that equalled or exceeded their congener 153 levels (Table 1). These were among the younger teachers (age 41 and under), who all worked at School A. The specific congener to PCB 153 ratios for the 18 teachers and the MGH referents were very similar for the congeners above PCB 74 (penta-chlorinated and above, Figure 2).

### Principal components analysis

**Figure 3:**
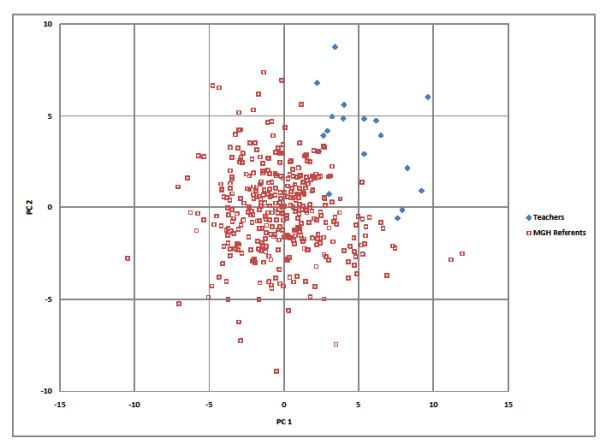
**PCA Score plot components 1 and 2 for teachers and MGH referents**.

Principal components analysis (PCA) was used to further assess the congener composition patterns among the 18 teachers and the MGH referents. In examining the summary table (Additional File 3: Results of principal components analysis), 33 of the 57 congeners loaded positively on principal components (PC) 1 and 2, including all the congeners lower than PCB 74. The differences between the component scores for the teachers and the MGH referents for PC1 and 2 were highly significant (p < 0.0001) by Wilcoxon rank-sum test. The score plot of the first two components shows a distinct difference between the populations, where the 18 teachers tend to have higher positive scores for both PC1 and PC2 (Figure 3).

## Discussion

Examining the pattern of congeners within the group of 18 teachers, there appears to be a relatively greater abundance of the lighter (PCB 6-74) congeners among the teachers from School A compared to the other two schools. The highest level of PCB 47 among teachers at schools B and C was 0.0800 ng/g, while five of the 10 teachers at School A exceeded this level. Details on the composition of each subjects' congener profile are presented in Additional File 4: Summary table of serum PCB whole weight (ng/g) by congener homolog group. All 18 teachers exceeded the median value of 0.0068 ng/g for the MGH referents, however. PCB 47 (2,2',4,4'-tetrachlorobiphenyl, CAS 2437-79-8) was reportedly present (0.07-0.14%) in the commercial formulation Aroclor 1254, which was commonly used as a plasticizer in caulking materials [11]. PCB 47 is among the more volatile congeners, vapor pressure 8.63 × 10^-5 ^mm Hg at 25 deg C [12], and it has a relative human accumulation factor of 0.03 [13] indicating that is among the less persistent PCB congeners. It's presence among the teachers may reflect their recent exposure, as estimated human half-life values for 0.2 to 5.5 years have been reported [11] for PCB 47. By comparison, the estimated half-life for PCB 153 ranges from 0.9 to 47 years.

In the case of the 18 teachers in the current study, there appeared to be a consistent difference in serum levels between the teachers and the MGH referents over the range of comparisons (Table 3). The difference is most apparent among congeners 6-74 (below the penta-chlorinated congeners). The relative human accumulation (RHA) [13] factors for these congeners are typically below 0.05, suggesting that they are more readily metabolized than the more persistent congeners (PCB 153 has a RHA of 1.1, for example). These are also the congeners with vapor pressures in the range of 10^-4 ^to 10^-8 ^mm Hg [tri- to hepta-chlorinated PCBs] that are sufficiently volatile to exist in the aerosol particle adsorbed and vapor phase [12]. This pattern of elevated serum congener levels may be attributed to the contribution of environmental sources, other than diet, to the overall PCB body burden in these teachers. The serum levels among the 18 teachers were elevated for all the congeners that have been identified as markers of non-dietary exposure, but only for one of the congeners higher than PCB 153 (Table 3). The ratios between the median congener levels among the teachers and the MGH referents were most prominently elevated among the lighter (PCB 6-74) congeners, but ratios of the measured dioxin-like PCB congeners (105, 118, 156, 157, 167, and 189) were all below 2, suggesting that occupancy of PCB-containing buildings did not substantially increase serum concentrations of these congeners.

Three of the men among the MGH referents reported that their occupation was "teacher", they were all aged 36 to 37 years. Their median total serum PCB was 2.69 ng/g (median MGH referents aged 30-39, 0.99 ng/g), and their median light congener level was 0.23 ng/g (MGH referents median 0.08 ng/g).

The PCA model further demonstrated significant differences between teachers and referents with regard to congener composition. The congeners that loaded most positively on PC1 and PC2 were PCB 6, 8, 16, 18, 25, 26, 31, 33, 37, 41, 44, 47, 49, 52, 66, and 95, confirming the differences observed in the other comparisons of the teachers and the MGH referents. Examining the score plot for the first two components, teachers had uniformly higher positive scores than the MGH referents.

A challenge in comparing serum PCB levels between groups is the selection of PCB congeners to use as exposure metrics [10,14,15]. The choice of congeners for comparison is limited by the fact that reference data frequently reports levels only for the most abundant congeners found in serum. For example, PCBs 28, 52, 101, 118, 138, 153 and 180 are frequently measured in serum to assess exposure. Grandjean et al. [16] chose congeners based upon their detectability in chemical analyses (PCBs 118, 138, 153, 170, 180, and 187), and PCBs 105 and 156 as they are mono-*ortho *congeners of toxicological interest. These sets of PCBs include several highly chlorinated congeners without vicinal hydrogens that tend to be highly persistent, and accumulate in the environment [17-19]. The major source of exposure to these congeners appears to be diet, particularly the consumption of fatty fish [20,21].

The World Health Organization (WHO) uses a set of 6 congeners as PCB indicators (28, 52, 101, 138, 153, and 180). Of these, 28 and 52 can be markers of indoor air contamination, while the others primarily have dietary sources [22]. For studies in the United States, the NHANES set of 35 PCB congeners is frequently used as a source of reference values. These congeners include those most frequently detected in the sampled population (138, 153, 180), and the set of 35 is weighted toward the hexa-chlorinated congeners and above (23 of the 35 are PCB 128 and higher).

Investigators [8,23-26] have proposed sets of specific PCB congeners that may serve as markers to distinguish between PCB exposures primarily from dietary sources, and the contribution of other exposure sources, such as employment in capacitor plants, where exposure occurred both by inhalation and dermal contact. In cases where inhalation of PCBs volatilized from contaminated outdoor sites or occupancy of contaminated buildings is likely to be a significant exposure pathway, lighter, lower chlorinated congeners (< PCB 74, tetra-chloro and below) would be expected to be more characteristic markers of exposure. Comparison of these sets of markers may also provide information about the temporality of exposure by separating more recent exposures from earlier exposures, as the presence of less-persistent, more readily metabolizable congeners suggests recent contact with sources of exposure. Wolff *et al. *[27] identified PCB congeners 28, 74, 118, 105, and 156 as unique markers of occupational exposure in a study of former capacitor plant workers. This was based upon the observation that levels of these congeners are low in the general population, and they are derived almost exclusively from the Aroclor mixtures used in the capacitor plants. De Caprio *et al. *[14] used polytopic vector analysis (PVA) to identify congener patterns in serum in a study of Akwesasne Mohawks with historical PCB exposure. Congeners 8, 18, 32/16, 31, 28, 33, 52, 44, 70, 66, 95, and 90/101 (primarily tri- and tetrachloro PCBs) were hypothesized to reflect recent inhalation exposure. Freels *et al. *[15] suggested that combinations of congener levels and their relative proportions should be considered relevant in tracking the source and pathway of PCB exposures. A recent animal study demonstrated that inhalation is a very efficient route of exposure and uptake, particularly for lower-chlorinated congeners [28].

We previously conducted a study comparing the congener-specific serum PCB concentrations between workers who removed old PCB caulk and the MGH referents. There were substantial differences between subject and referent mean and median serum levels for PCB 6, 16, 26, 33, 37, 41, 70, 97, and 136 [29]. For these congeners, the subject mean (and median) exceeded the reference mean (and median) by a factor of 5 or more. The congeners found to be most substantially elevated among the construction workers were those rapidly eliminated from the body, typically with relative human accumulation (RHA) factors from 0.001 to 0.07 [13] and short apparent human half lives, e.g. 0.02 years, approximately 1 week for PCB 33 [11]. Notably, the NHANES data does not include any of these congeners (6, 16, 26, 33, 37, 41, 70, 97, and 136).

While our study provides evidence that the 18 teachers have elevated serum levels of certain light PCB congeners, suggesting an environmental source, several limitations must be considered in interpreting the data. First, the comparisons with the NHANES data are restricted by the dissimilarity in the sets of congeners reported by the two laboratories and differences in the laboratory methods used. The main reason for this dissimilarity apparently is that the analytical strategy of the CDC laboratory is designed for serum analysis, where a high number of light, lower chlorinated congeners would not be expected. In contrast, our laboratory chose the list of target congeners considering that it would be conducting analysis for a number of studies, some of which would include air samples, as well as biological samples. Therefore more low chlorinated (more volatile) congeners were included in our analytical strategy. This mixture of congeners is examined in all matrices, including air samples and blood serum.

Another limitation was that the MGH study included only men, while the majority (13 of 18) of our teachers were women. The 2003-4 NHANES data indicated that total (sum of 35 congeners) levels were slightly higher in men than in women [30], so the difference we saw between the teachers and the MGH referents may underestimate the true difference. In addition, we cannot rule out the possibility that there are differences between men and women in elimination (biotransformation) of lower molecular weight PCBs. We were unable to recruit a referent group of teachers who worked only in schools built after 1980, which would be expected to be free of PCB-containing building materials. Comparing the MGH referents with NHANES data for the same age strata (20 to 51) for the 33 congeners reported in common, the MGH subjects have significantly elevated levels for the sum of the 33 congeners compared to the NHANES, however the levels of 5 of the 6 congeners in the PCB 6-74 range are significantly lower for the MGH subjects than the age-comparable NHANES values. Both the 18 teachers and the MGH referents have lower levels of the 6 light congeners than age-comparable NHANES results. While these levels may actually be lower, it is also possible that inter-laboratory differences may contribute to these findings.

Another factor we considered in the comparison between the teachers and the MGH referents is the lack of information we have about the contact the referents may have had with non-dietary sources of PCBs. The 18 teachers were all sampled at the end of the last day of school in 2009, but the MGH referents were sampled during weekday clinic visits, and we have no information, for example, on how recently or how much time they spent in a PCB-containing building. Finally, ours was a pilot study, limited by the small sample size (18 teachers) and the fact that we did not have access to characterize the indoor environments of the schools by air sampling. We cannot explain, for example, the differences in serum levels of light congeners (e.g., PCB 47) seen between the teachers at school A and the teachers from other schools. A larger study that includes a comprehensive evaluation of potential PCB sources associated with the buildings (caulk, fluorescent light ballasts, ceiling tiles, carpet adhesives, and paints) is clearly needed.

## Conclusions

Despite the limitations, this study provides evidence that occupants of PCB-containing buildings have elevated serum levels of several PCB congeners. The nature of the difference between the teachers and the referents is not apparent by comparison with the NHANES reference values which are weighted toward measuring the more persistent, highly chlorinated congeners. The congener profile for the 18 teachers, however, is significantly enriched with lighter congeners, including those that have been previously identified as coming from non-dietary sources. These findings show the importance of analyzing a wide range of PCB congeners when seeking to identify the possible sources of human exposures. In view of the growing concern about occupancy of PCB-containing buildings, investigators should assess as full a profile of congeners as feasible in environmental and biological samples to fully characterize the nature and sources of PCB body burdens.

## List of Abbreviations

EPA: Environmental Protection Agency; NHANES: National Health and Nutrition Examination Survey; ng/g: nanogram per gram; pg/g: picogram per gram; PCA: principal components analysis; PCB: polychlorinated biphenyls.

## Competing interests

The authors declare that they have no competing interests.

## Authors' contributions

RFH designed the study and was responsible its overall conduct; JDM was involved in the analysis and interpretation of the data and participated in the manuscript preparation; LA conducted the sample analysis and data compilation. All authors read and approved the final manuscript.

## Supplementary Material

Additional file 1 **Details of the Quality Assurance and Quality Control procedures**. Click here for file

Additional file 2 **Characteristics of teacher study subjects**. Click here for file

Additional file 3 **Summary table results of Principal Components Analysis**. Click here for file

Additional file 4 **Summary table of teacher serum PCB level by school, age and congener homolog group**. Click here for file
