# Supplementary material for: Serum PCB levels and congener profiles among teachers in PCB-containing schools: a pilot study
Source: Environ Health. 2011 Jun 13;10:56. doi: 10.1186/1476-069X-10-56 (PMC3136408; doi:10.1186/1476-069X-10-56)
Supplement: Additional file 1 — Details of the Quality Assurance and Quality Control procedures. [file 1476-069X-10-56-S1.PDF]

Quality Assurance and Quality Control. Strict quality control and quality assurance procedures were followed during sample analyses. Background contamination determined by the ΣPCB congeners in procedural blank was 0.37 ng/g. Analytical accuracy, precision, and extraction efficiency were evaluated by the analyses of standard reference material (SRM 1589, NIST *Aroclor 1260 in Human Serum*), one pair of matrix spike samples (aliquots of pooled serum spiked with 0.17 ng/g of each target PCB congener), and two surrogate compounds added to each sample prior to extraction.

The Mean (± SD) percent recoveries of two surrogate compounds (PCB#30 and PCB# 204) added to all samples to monitor extraction efficiency were 95% (± 2) and 88% (± 5), respectively.

The Mean (± SD) percent recoveries of all target PCB congeners in Matrix spike and Matrix spike duplicate samples were 91% (±4.7) and 88% (±4.5) respectively. The relative percent difference between concentrations of all target PCB congeners in two matrix spike samples, expressed as mean RPD (± SD) was 3.8% (±2.3).

The method detection limits (MDL) for targeted individual PCB congeners ranged from 0.002 to 0.036 ng/g serum, with most MDLs < 0.01 ng/g serum.

The laboratory has been successfully participating in International Inter-calibration, AMAP Ring Test for PCBs and OCs in Plasma, sponsored by AMAP (Arctic Monitoring and Assessment Program) and organized by Quebec National Institute of Public Health, Canada.
