# Supplementary material for: Serum PCB levels and congener profiles among teachers in PCB-containing schools: a pilot study
Source: Environ Health. 2011 Jun 13;10:56. doi: 10.1186/1476-069X-10-56 (PMC3136408; doi:10.1186/1476-069X-10-56)
Supplement: Additional file 2 — Characteristics of teacher study subjects. [file 1476-069X-10-56-S2.PDF]

Additional file 2: Characteristics of teacher study subjects

Subject	Weight (pounds)	Age (years)	Gender	Diet
6	130	33	f	Canned tuna, shrimp, lobster, whitefish 1-3X/month
12	115	35	f	All never
9	132	37	f	Canned tuna, dark fish, white fish 1-3X/month
8	228	41	m	Canned tuna, dark fish, shrimp, lobster, white fish 1-3X/month
11	187	41	f	All never
16	135	46	f	All never
15	190	47	f	Shrimp, lobster, whitefish 1-3X/month
4	157	48	f	All never or <1X/month
3	150	49	f	Canned tuna, shrimp, lobster, whitefish 1-3X/month
5	130	52	f	Canned tuna 1-3X/month, whitefish, liver both beef and chicken, 2-4X/wk
10	198	54	m	All never
17	161	56	f	Dark fish, shrimp, lobster, white fish 1-3X/month
2	250	56	m	Whitefish 2-4X/wk, liver 1-3X/month
14	215	59	m	Canned tuna, dark fish, white fish, liver(beef) 1-3X/month
7	nr	60	f	Dark fish, shrimp, lobster, white fish 1-3X/month
1	210	62	f	Whitefish1X/wk
13	240	62	m	Canned tuna, shrimp, lobster1-3X/month, dark fish, white fish 1X/wk
18	295	64	f	Dark fish, white fish 2-4X/week, Shrimp, lobster 1-3X/week
