# Supplementary material for: Serum PCB levels and congener profiles among teachers in PCB-containing schools: a pilot study
Source: Environ Health. 2011 Jun 13;10:56. doi: 10.1186/1476-069X-10-56 (PMC3136408; doi:10.1186/1476-069X-10-56)
Supplement: Additional file 3 — Summary table results of Principal Components Analysis. [file 1476-069X-10-56-S3.PDF]

Additional file 3 --. Results of principal components analysis

Congener	PC1	PC2	PC3	PC4	PC5	PC6
PCB6	0.054	0.139	-0.276	0.159	0.171	0.091
PCB8	0.153	0.114	-0.159	-0.120	-0.002	0.056
PCB18	0.060	0.093	-0.046	-0.121	-0.188	0.042
PCB16	0.056	0.059	-0.079	0.018	0.158	-0.385
PCB26	0.101	0.112	-0.203	-0.142	0.020	0.032
PCB25	0.090	0.072	-0.058	-0.204	-0.201	-0.022
PCB31	0.133	0.108	-0.194	-0.020	0.063	0.124
PCB28	0.207	0.047	-0.043	0.060	-0.195	0.022
PCB33	0.139	0.166	-0.153	-0.179	0.105	-0.019
PCB52	0.157	0.031	0.128	0.064	0.149	-0.243
PCB49	0.099	0.070	0.003	-0.182	-0.232	-0.366
PCB47	0.045	0.088	-0.179	0.023	0.091	-0.251
PCB44	0.109	0.081	0.010	0.040	0.067	-0.048
PCB41	0.136	0.162	-0.218	-0.048	0.082	0.132
PCB37	0.178	0.186	-0.188	-0.160	0.010	0.042
PCB95	0.239	0.008	0.072	0.157	0.023	-0.150
PCB66	0.259	0.032	0.060	0.113	0.026	-0.135
PCB74	0.190	-0.026	-0.136	0.221	-0.209	0.047
PCB70	0.112	0.093	0.033	-0.065	0.004	-0.214
PCB84	0.113	0.049	0.003	0.072	0.201	-0.299
PCB60	0.136	-0.026	0.108	0.151	-0.191	-0.065
PCB99	0.229	-0.115	-0.059	0.203	-0.004	0.037
PCB101	0.234	0.022	0.135	-0.022	0.133	-0.058
PCB97	0.144	0.121	-0.110	-0.085	0.117	0.076
PCB87	0.200	0.063	0.043	-0.090	0.023	-0.047
PCB136	0.137	0.145	-0.173	-0.040	0.124	0.137
PCB77_110	0.185	0.062	0.111	-0.008	-0.060	0.274
PCB151	0.186	0.018	0.236	-0.123	-0.070	0.056
PCB135	0.145	-0.003	0.180	-0.082	0.201	0.068
PCB149	0.176	0.055	0.243	-0.131	-0.096	0.174
PCB118	0.241	-0.085	-0.053	0.224	-0.145	0.062
PCB146	0.018	-0.027	0.056	0.093	-0.108	0.094
PCB105_141	0.219	-0.081	0.068	0.268	-0.051	0.056
PCB138	0.061	-0.162	-0.018	0.292	0.074	0.023
PCB187	-0.039	0.214	0.291	0.111	0.040	-0.024
PCB183	0.042	0.106	0.175	-0.048	0.249	0.049
PCB128	0.122	0.003	0.145	-0.061	0.100	0.070
PCB174	0.147	0.047	0.188	-0.233	-0.047	0.232
PCB167	0.026	-0.012	-0.094	0.165	0.093	0.272
PCB177	0.029	0.014	0.299	0.136	0.134	0.066
PCB157_201	-0.009	0.053	-0.057	0.213	0.182	0.002

PCB171	-0.072	0.163	0.158	-0.012	0.297	0.063
PCB156	-0.083	0.051	-0.193	0.209	-0.159	0.016
PCB180	-0.166	0.288	0.074	-0.020	-0.068	-0.041
PCB170	-0.187	0.222	-0.014	0.052	0.060	0.001
PCB199	-0.063	0.322	0.088	0.149	-0.135	-0.011
PCB196_203	-0.057	0.325	0.089	0.123	-0.143	-0.002
PCB189	-0.086	0.122	-0.097	0.110	0.220	0.176
PCB195	-0.088	0.180	0.061	0.135	0.044	0.124
PCB194	-0.102	0.330	0.052	0.044	-0.169	-0.014
PCB206	0.007	0.285	0.034	0.183	-0.260	-0.016
PCB209	-0.009	0.171	0.042	0.184	0.102	-0.083
Eigenvalue	9.26	6.49	3.59	2.91	2.48	2.24
Proportion of Variance	0.18	0.12	0.07	0.06	0.05	0.04
Cumulative Variance	0.18	0.30	0.37	0.43	0.48	0.52
p-value*	<0.0001	<0.0001	0.02	<0.0001	0.10	0.04

*Wilcoxon rank-sum test comparing component scores between Teachers and MGH referents
