# Supplementary material for: Serum PCB levels and congener profiles among teachers in PCB-containing schools: a pilot study
Source: Environ Health. 2011 Jun 13;10:56. doi: 10.1186/1476-069X-10-56 (PMC3136408; doi:10.1186/1476-069X-10-56)
Supplement: Additional file 4 — Summary table of teacher serum PCB level by school, age and congener homolog group. [file 1476-069X-10-56-S4.PDF]

Additional file 4 – Summary table of serum PCB whole weight (ng/g) by congener homolog group

	Subject age	sum mono, di, tri (PCB 6-37)	sum tetra (PCB 41-74)	sum penta (PCB 84-118)	sum hexa (PCB 128-167)	sum hepta (PCB 170-189)	sum octa, nona (PCB 194-209)	∑ PCB congeners
School A								
12	35	0.0675	0.1644	0.1151	0.1953	0.1088	0.0940	0.7409
8	41	0.0449	0.1648	0.0553	0.1625	0.1064	0.0521	0.5800
11	41	0.0722	0.2224	0.1329	0.3307	0.2206	0.1101	1.0868
16	46	0.0514	0.1016	0.1363	0.3903	0.4819	0.3315	1.4919
15	47	0.0615	0.2038	0.3025	0.5450	0.2164	0.0975	1.4266
4	48	0.0635	0.2131	0.2147	0.6750	0.5357	0.3218	2.0218
3	49	0.0681	0.1058	0.1078	0.3310	0.2267	0.1225	0.9609
10	54	0.0841	0.2049	0.1652	0.7093	0.4923	0.2622	1.9171
17	56	0.0754	0.2097	0.2552	0.8710	0.6815	0.3542	2.4450
1	62	0.0744	0.2046	0.2550	0.7313	0.4057	0.2073	1.8782
School B								
6	33	0.0691	0.0916	0.1710	0.2937	0.1228	0.0584	0.8046
9	37	0.0446	0.0739	0.1637	0.2655	0.1199	0.0695	0.7341
2	56	0.0379	0.0790	0.1558	0.5205	0.3576	0.2263	1.3772
14	59	0.0568	0.1835	0.1784	0.5435	0.4452	0.2726	1.6770
School C								
5	52	0.0696	0.1243	0.2199	0.7637	0.7602	0.5279	2.4646
7	60	0.0565	0.1349	0.2169	0.8887	0.5931	0.2823	2.1714
13	62	0.1091	0.2771	0.6815	1.8305	0.9860	0.4886	4.3708
18	64	0.1972	0.4661	1.6262	1.9524	0.7198	0.3569	5.3185
